# Effect of Single Dose Administration of Methylsulfonylmethane on Oxidative Stress Following Acute Exhaustive Exercise

**Published:** 2013

**Authors:** Babak Nakhostin-Roohi, Zahra Niknam, Nasrin Vaezi, Sadollah Mohammadi, Shahab Bohlooli

**Affiliations:** a *Department of Exercise Physiology, Ardabil Branch, Islamic Azad University, Iran. *; b *Department of Exercise Physiology, University of Mohaghegh-Ardabili, Ardabil, Iran. *; c *Department of Pharmacology, School of Pharmacy, Ardabil University of Medical Sciences, Ardabil, Iran.*

**Keywords:** MSM, Antioxidant, Protein carbonylation, GSH, Uric acid, Bilirubin, TAC, MDA

## Abstract

Methylsulfonylmethane (MSM) is a sulfur-containing compound commonly found in diet and known to reduce oxidative stress. This trial was conducted to determine whether single dose supplementation with MSM attenuates post-exercise oxidative stress in healthy untrained young men. Sixteen untrained men volunteered for this study. Participants were randomized in a double-blind placebo-controlled fashion into 2 groups: Methylsulfonylmethane (MSM) (n = 8) and placebo (n = 8). The participants took supplementation or placebo before running on treadmill for 45 min at 75% VO_2max_. The MSM supplementation was prepared in water as 100 mg/ kg body weight. The placebo group received water. Serum Malondealdehyde (MDA), uric acid, bilirubin, protein carbonyl (PC) and plasma vitamin E levels were determined as the markers of oxidative stress. Plasma GSH (reduced Glutathione) and total antioxidant capacity (TAC) were measured as markers of plasma antioxidant system. MSM supplementation successfully lowered serum PC 2 and 24 h after exercise. Plasma TAC in MSM group was higher at 24 h after exercise. Serum level of uric acid and bilirubin were significantly low immediately after exercise in MSM supplemented group. There was no significant difference between groups in terms of plasma GSH level. These results complement earlier studies showing anti-oxidant effect of MSM and suggest that single dose oral supplementation with MSM lowers exercise induced oxidative stress in healthy untrained young men, but is not adequate to significantly affect plasma GSH level.

## Introduction

The energy demand during physical exercise causes an increased oxygen uptake and supply to active tissues, which may elevate the rate of reactive oxygen species (ROS) generation and production of free radicals (1). Oxidative stress is a condition in which the existing balance between free radicals production and their subsequent amelioration via the antioxidant defense system becomes skewed in favor of free radical expression (2, 3). During exhaustive exercise, antioxidant defense system is likely to be compromised by insufficiency of endogenous antioxidants. Therefore, given the potential involvement of ROS in detrimental cellular processes, research has focused on the possible beneficial effects of antioxidant consumption (1). Supplementation with antioxidants may help to attenuate the damage of the body induced by oxidative stress (4). Several studies have reported that supplementation with antioxidants is a good alternative for reducing the damage caused by exercise in athletes or non-athletes (5). Although antioxidant intake through foods and low to moderate dose nutritional supplements, is generally considered to provide health-enhancing benefits, higher-dose supplemental antioxidant intake is somewhat controversial (6). MSM is a sulfur-containing compound with low toxicity and is found in a wide range of human foods including fruits, vegetables, grains, and beverages (7, 8). Recently, MSM has received wide attention as a dietary supplement in the treatment of osteoarthritis (7, 9, 10). It was shown that MSM is effective in seasonal allergic rhinitis (11), interstitial cystitis (12), autoimmune disease (13), cancer chemoprevention (14, 15) and offers anti-inflammatory and anti-oxidant effects (13, 16, 17).

Our previous study showed that the administration of MSM for 10 days to young healthy men is able to significantly lower the known markers of oxidative stress such as Malondialdehyde and protein carbonyl following exhaustive exercise (16). Therefore, this study was undertaken to investigate the possible effects of acute single dose administration of MSM on some markers of oxidative stress following acute exercise in untrained healthy young volunteers.

## Experimental

*Participants*

Sixteen untrained healthy men (mean ± SD: age, 19.815 ± 1.35 years; height, 173.6 ± 5.22 cm; body mass [BMI], 24.015 ± 2.35 Kg/m^2^) volunteered for this study. Each participant completed a pre-exercise health status questionnaire. None of the participants reported: (a) a history of medical or surgical procedures that might have significantly affected the study outcome, including cardiovascular disease or metabolic, renal, hepatic, or musculoskeletal disorders; (b) use of smoking or any medication that might have significantly affected the study outcome; (c) use of any nutritional supplements (*i.e*., creatine, protein drinks, amino acids, and vitamins) in the 8 weeks before the beginning of the study; or (d) participation in another trial or ingestion of another investigational product within 30 days before screening and enrollment. All the participants were informed of the aim of the study and a written informed consent was obtained. The protocol of the study was approved by the university ethics committee in accordance with the Helsinki Declaration.

*Experimental design*

All procedures were completed at the laboratory of Ardabil Sport Medicine Committee. Two weeks prior to main test, participants underwent Bruce test on treadmill for determining their VO_2max_ (maximal oxygen consumption). Body fat composition was estimated using the sum of three skin-folds (chest, abdomen, thigh) as outlined elsewhere (18). Participants were randomized in a double-blinded placebo-controlled fashion into 2 groups: Methylsulfonylmethane (MSM) (n = 8) and placebo (n = 8). On day of the test, they arrived at laboratory after an overnight fasting. A baseline blood draw was taken, and then they were allowed to take breakfast. When the above tests were completed, the drink, either placebo (200 mL water) or the MSM supplement (100 mg/kg methylsulfonylmethane in 200 mL water [adapted from the work of Kim *et al*. (7)]), was given. After 2 h rest, the second blood samples were collected. Following a 10 min warm up consisting of running at 50% VO_2max_ (5 min) and stretching (5 min), participants ran on treadmill for 45 min at 75% VO_2max_. Final speed of running is enhanced 0.5 km/h every 2 min until exhaustion. The participants were allowed to consume water *ad libitum *throughout the exercise and afterwards. Subsequent blood samples were taken immediately, and at 2 and 24 h after exercise.

*Blood sampling and analysis*

Approximately, 10 mL of blood was withdrawn at each time point. Three ml of blood was placed in heparinized tubes and centrifuged at 3000 rpm for 10 min at 4°C. Plasma was transferred to microtubes and stored at -80°C for subsequent analysis. Rest of the blood was allowed to clot and centrifuged at 5000 rpm for 10 min. Serum was removed and aliquoted in 0.2 mL volumes and stored at -80°C until analysis.

Total anti-oxidant capacity (TAC) was measured using ferric reducing ability of plasma (FRAP) method (19).

Serum Malondealdehyde (MDA) was determined by method of Mateos *et al *(20). In brief, an aliquot of 100 μL of serum was placed in a 1.5 mL microtube and 20 μL of 6 M NaOH were added. Alkaline hydrolysis of protein bound MDA was achieved by incubating this mixture in a 60°C water bath for 30 min. Protein was precipitated with 50 μL of 35 % (v/v) perchloric acid, and the mixture was centrifuged at 2800 × g for 10 min. A 100 μL volume of supernatant was transferred to a microtube and mixed with 100 μL of 2, 4 nitrophenylhydrazine (DNPH) prepared as a 5 mM solution in 2 M hydrochloric acid. Finally, this reaction mixture was incubated for 30 min at room temperature protected from light. An aliquot of 20 μL of the reaction mixture was injected onto HPLC system equipped with C18 column (4.6 × 250 mm, 5 μ).

Protein Carbonyls (PC) content of serum were measured according to the method described elsewhere (19) with slight modifications as reported by Baltacioglu *et al*. (22) using 2,4 nitrophenylhydrazine (DNPH) reagent. The carbonyl content was calculated from peak absorption (360 nm) using an absorption coefficient (e) of 22,000 M-1cm^-1^. Each sample was read against the control sample. The PC content was expressed as concentration (μmol/L) in serum.

Plasma GSH determination was performed as described by Giustarini *et al*. (23) with slight modification using ion exchange chromatography. Briefly, 100 μL of plasma samples were diluted with an equal volume of TCA [5% (w/v) final concentration] and centrifuged at 15,000 × g for 2 min. After supernatant alkalization, samples were reacted with an equal volume of 2,4-dinitrofluorobenzene (FDNB) solution [1.5% (v/v) in ethanol] for 3 h at room temperature in the dark. After acidification with 10 μL HCl [37% (v/v) initial concentration], 20 μL of sample was loaded on HPLC.

Plasma vitamin E level was determined by method of Catignani (24) with slight modification. In a brief, 100 μL of plasma was placed in a microtube, 50 μL ethanol and 50 μL tocopherol acetate as internal standard were added to the tube and vortexed. Two hundred microliter of *n*-hexane was added to the mixture and vortexed for 45 sec. One hundred microliter of the organic layer was transferred to clean tube and dried under N2 stream. The residue was dissolved in 200 μL methanol. 100 μL of methanolic solution was injected onto HPLC system.

Serum uric acid and bilirubin were determined using commercially available kits (DarmanKav Co, Iran) and double beam spectrophotometer (T80+, PG instruments, England).

*Statistical analysis*

Results are expressed as mean ± standard error of mean (SEM). Data were analyzed for time and group intervariability using two way repeated measures analysis of variance (two-way ANOVA). When appropriate, significant differences among means were tested using Bonferroni post hoc test. Between groups comparison for subject characteristics was done using unpaired t-test. Differences between groups were considered to be significant when p < 0.05.

## Results and Discussion

This study examined whether single dose (100 mg/kg) oral supplementation with MSM prior to exercise was able to exert anti-oxidant effect following exhaustive acute exercise in healthy untrained young men. To our knowledge, these findings are the first to show that single dose oral supplementation with MSM lowers serum protein carbonyl content, uric acid and bilirubin after exercise. In addition, total anti-oxidant capacity of plasma was increased at 24 h after exercise in MSM treated group.

*Participants’ characteristics*

The characteristics of participants including age, weight, percent body fat, BMI and preliminary VO2max are summarized in Table 1.

**Table 1:** Comparison of subjects’ characteristics in MSM and placebo groups

	MSM	Placebo	*P* value*
Age (years)	20.1 ± 1.4	19.5 ± 0.8	0.69
Height (cm)	170.9 ± 5.2	176.3 ± 5.2	0.48
Weight (kg)	70.9.± 8.1	74.1 ± 10.5	0.81
Body mass index (kg.m^-2^)	24.2 ± 2.4	23.9 ± 3.3	0.94
Body fat (%)	9.6 ± 3.7	9.8 ± 3.9	0.97
VO_2max_ (ml.kg^-1^.min^-1^)	39.9 ± 3.3	40.0 ± 4.4	0.98

Values are mean ± SEM (n=8); VO_2max_ maximal oxygen consumption. * Mean values was not significantly different between MSM and Placebo

*Dietary analysis*

Dietary analysis revealed no differences in energy, protein, fat, carbohydrate and antioxidant vitamins intake between groups throughout the study (Table 2).

**Table 2:** Dietary Analysis in MSM and placebo groups

	Placebo	MSM	P-value*
Energy (Kcal)	2009 ± 241	1994 ± 231	0.992
Carbohydrate (g)	291 ± 62	287 ± 64	0.996
Protein (g)	98 ± 28	74 ± 19	0.751
Fat (g)	50 ± 13	62 ± 18	0.800
Vitamin A(mg)	296 ± 115	326 ± 181	0.719
Vitamin C (mg)	38 ± 18	37 ± 22	0.928
Vitamin E (mg)	14 ± 9	17 ± 12	0.416

Data are means ± standard deviations of mean (n=8). * Mean values was not significantly different between MSM and Placebo.

*MDA level*

Single dose administration of MSM had significant effect on serum MDA level (p = .02). Post hoc analysis showed significant difference on MDA level at pre-exercise (p = 0.036) and 2 h after exercise (p = 0.034) when comparing P and M groups (Table 3). There was not a significant elevation on MDA level after exercise in both groups, nevertheless a meaningful decrease in MDA levels at pre-exercise and 2 h after exercise was observed in MSM supplemented group. The increase in lipid peroxidation by-products following single bout of exercise was confirmed by many authors (25-27). However, result of current study failed to show any significant increase in serum MDA level following exercise. In accordance, the majority of studies which utilized specific measure of MDA have noted no increase in MDA following exercise (2). This result could be due to insufficiency of the exercise protocol used to induce adequate oxidative stress which was marginally unable to increase the serum MDA as high as expected from our previous study (16). Nevertheless, MSM supplementation caused a significant decrease in MDA at pre and 2 h after exercise. A decrease in serum MDA and PC levels could be explained by direct radical scavenging (28) or inhibitory effect of MSM on free radicals generation (29).

**Table 3:** Values of GSH, Uric Acid and Vitamin E in serum or plasma of subjects before and after acute bout of exercise in Placebo and Methylsulfonylmethane supplemented (MSM) groups

	Group	Baseline (-2 h)	Pre	PE	2 PE	24 PE
MDA (µM/L)	P	2.53±0.18	2.76±0.23	3.1±0.43	2.72±0.32	2.69±0.18
	M	2.37±0.29	2.00±0.18^*^	2.55±0.19	1.95±0.11^*^	2.29±0.22
GSH (µM/L)	P	9.43±0.53	9.78±0.88	8.24±0.22	8.86±0.24	8.82±0.17
	M	8.75±0.33	9.42±0.46	8.35±0.18	8.84±0.43	9.80±0.71
Uric Acid (mg/dL)	P	2.45±0.15	2.32±0.30	3.16±0.16^+^	2.93±0.15^+^	2.53±0.11
	M	2.06±0.11	2.40±0.11	2.63±0.06^*^	2.73±0.09^+^	2.59±0.17^+^
Vitamin E (µg/mL)	P	10.98±0.53	10.94±0.72	10.76±0.83	12.39±0.74	11.26±0.97
	M	9.87±0.49	9.98±0.83	10.69±0.96	9.46±0.31^*^	10.13±0.27

Values are means ±SEM (n=8)* Mean value was significant vs. placebo group at the same time point; + Mean value was significant vs. Pre value; Pre: pre exercise; PE: Post Exercise; GSH: Reduced Glutathione;

*GSH level*

Plasma GSH level was not affected significantly by single dose MSM supplementation or exercise. The results of two factor ANOVA did not reveal any significant treatment effect (p = 0.828), time effect (p = 0.062), or treatment by time interaction (p = 0.64). Although, Maranon et al reported that chronic administration of MSM to jumping horses for 6 weeks was able to increase plasma GSH level (30) which was also confirmed with our previous study (16) , it seems that single dose supplementation prior to exercise was not sufficient to elevate plasma GSH level in men.

*Protein carbonyl (PC) content*

**Figure 1:**
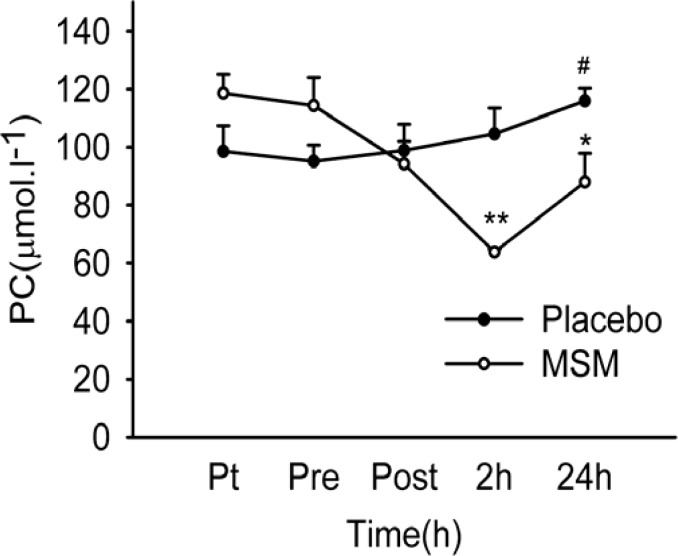
Serum PC content after acute bout of exhaustive exercise under MSM or placebo administration. Values represent means ± SEM (n = 8). ** p < 0.001 significant difference in change in MSM vs. Placebo; * p < 0.05 significant difference in change in MSM vs. Placebo; # p < 0.05 significant difference from pre-exercise values, same treatment. Pt pre-treatment (base line), Pre pre-exercise, Post post-exercise

As depicted in Figure 1, PC content of serum increased significantly at 24 h after exercise above pre-exercise values (p = 0.007) in placebo group. There was also a significant decline of serum protein carbonyl level at 2 and 24 h after exercise in MSM group. The post hoc test revealed significant differences between 2 and 24 h after exercise versus pretreatment values (p < .001 and p = 0.018, respectively) in MSM group. Again, post hoc analysis showed significant differences on PC level at 2 and 24 h after exercise (p < 0.001 and p = 0.012, respectively) comparing placebo and MSM groups (Figure 1). In this study, serum PC showed significant elevation above pre-exercise values at 24 h after exercise only in placebo group. The enhancement of protein carbonylation following training or single exercise was observed by many investigators (31-33). Our study has demonstrated that MSM administration caused a significant decrease in PC level at 2 and 24 h after exercise. The preventive effect of antioxidant supplementation on oxidative damage to proteins has been reported previously (34-36). Accordingly, in agreement with work of Nakhostin-Rooh *et al. *(16) the result of current study showed that single dose MSM supplementation was able to attenuate protein carbonylation.

*Plasma total antioxidant capacity (TAC)*

**Figure 2:**
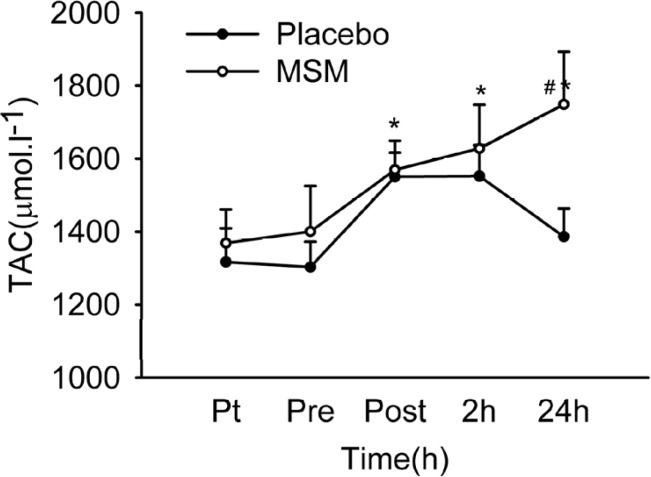
Plasma TAC level after acute bout of exhaustive exercise under MSM or placebo administration. Values represent means ± SEM (n = 8). * p < 0.01 significant difference from pre exercise values, same treatment; # p < 0.05 significant difference in change in MSM vs. Placebo group. Pt pre-treatment (base line), Pre pre-exercise, Post post-exercise

Total antioxidant capacity of plasma (Figure 2) was increased significantly immediately and 2 hours after exercise above pre-exercise values in placebo group, and at 2 and 24 h after exercise in MSM group. A significant difference was detected at 24 h values (p = 0.017) when comparing placebo and MSM groups. TAC was maintained high at all-time points after exercise in MSM group but declined at 24 h after exercise in placebo group. Increase in total antioxidant capacity following exercise were reported (2). In accordance, our study demonstrates an increase in plasma TAC level immediately and 2 h post exercise. Moreover, MSM supplementation maintained TAC elevation at 24 h after exercise. Similarly, other studies have also reported the enhancing effect of anti-oxidant supplementation on plasma total anti-oxidant capacity (36).

*Serum uric acid level*

Serum level of uric acid increased after exercise in both groups which was significantly higher in placebo group immediately after exercise compared with the MSM group (Table 3). Serum uric acid increased significantly above pre-exercise values immediately and 2 hours after exercise (p = 0.001 and p = 0.042, respectively) in placebo group. There was also a significant elevation in uric acid level at 2 and 24 h after exercise (p = 0.003 and p = 0.01, respectively) above pre-exercise values in MSM group. The post hoc analysis showed a significant difference between placebo and MSM groups at post-exercise time points (p = 0.039). MSM supplementation prior to exercise influenced urate metabolism significantly. The increase in serum urate level after exercise has been confirmed by several studies (37, 38). Increase in serum urate could be attributed to degradation of adenine nucleotides and transformation of xanthine dehydrogenase into xanthine oxidase, possibly through the oxidation of free sulfhydryl groups (36). Exhaustive exercise induces degradation of adenine nucleotides in skeletal muscle and produces hypoxanthine. Hypoxanthine released into blood (29) is oxidized to urate by liver (37, 38) or plasma (38) xanthine oxidase, which subsequently leads to an increased serum urate. This study is in agreement with an increase in serum urate level after exhaustive exercise, but the increase depends on MSM supplementation prior to exercise, since serum urate elevation in the supplemented group was not as high as the placebo. In parallel, the attenuating effect of anti-oxidant supplementation on serum uric acid following exercise has been reported by others (37).

*Bilirubin level*

**Figure 3:**
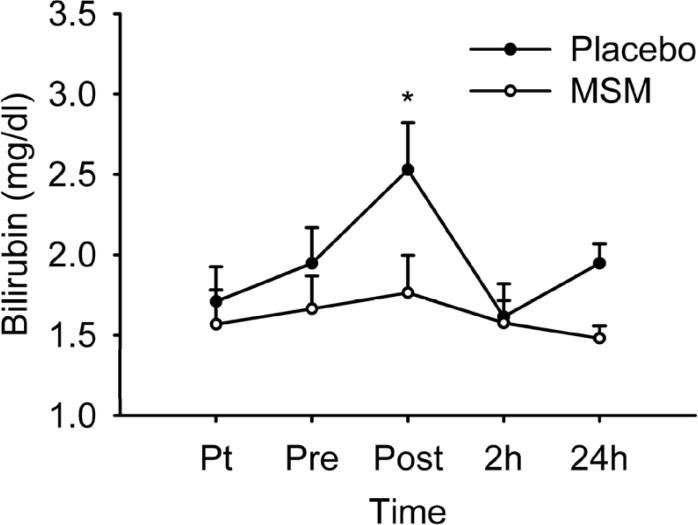
Serum total bilirubin level after acute bout of exhaustive exercise under MSM or placebo administration. Values represent means ± SEM (n = 8). * p < 0.05 significant difference in change in Placebo from MSM and pre exercise values. Pt pre-treatment (base line), Pre pre-exercise, Post post-exercise

Figure 3 represents serum level of total bilirubin. There was no significant treatment main effect (p = 0.198) or treatment by time interaction (p = 0.190), but a significant time effect (p = 0.005) was detected. Post hoc analysis revealed only a significant elevation in serum bilirubin level immediately after the exercise above pre-exercise values (p = 0.005) in placebo group. A significant difference was also detected at post-exercise values (p = 0.017) when comparing placebo and MSM group, showing a low level of serum bilirubin in MSM group. Serum bilirubin level also increased immediately after exercise in placebo group, but not in MSM group. Some studies reported the elevation of serum bilirubin level after exercise (40).

Bilirubin is reduced form of biliverdin which is metabolic product of heme degradation (41). Main enzyme responsible for heme metabolism is heme oxygenase, which exists in several isoforms. Heme oxygenase-1 (HO^-1^), the inducible form, shows increased activity during oxidative conditions such as acute exercise (42). During oxidative stress, heme is released from intracellular heme containing proteins such as myoglobin and hemoglobin. HO^-1^ plays a protective role by degrading heme as a pro oxidant and also by providing bilirubin (39). Bilirubin is the potent physiological antioxidant which is produced by HO^-1^ to prevent or counteract oxidative stress-mediated injury (23). It is likely that MSM supplementation was able to alleviate oxidative stress and decrease the HO^-1^ activity. As a result, the supplemented group showed low level of serum bilirubin. This finding was in correlation with attenuating effect of MSM on serum uric acid following exercise.

*Plasma vitamin E*

There was no significant difference within group on vitamin E levels in placebo or MSM group. A significant increase was measured for vitamin E level in placebo group when comparing with MSM at 2 h after exercise (p = 0.043) (Table 3). Increase in plasma vitamin E after exercise is attributed to lipid mobilization and increased secretion of RRR-*α*-tocopherol from peripheral tissues thus raising the corresponding plasma level (39, 43). It seems that MSM supplementation may blunt the increase in plasma vitamin E level. This is in parallel with decrease in serum PC levels 2 h after exercise.

## Conclusion

The present study showed that exercise protocol used was somehow able to induce oxidative stress in terms of PC, bilirubin and uric acid on healthy untrained men, but the protocol failed to significantly increase the serum level of MDA.

Single dose oral supplementation with MSM had some alleviating effects on protein carbonylation and may increase plasma total anti-oxidant capacity and also exert alleviating effect on MDA, serum uric acid and bilirubin levels following acute exercise. Although, it seems that acute administration of MSM prior to exercise may alleviate some markers of oxidative stress, but it is not adequate to increase plasma GSH level. Nevertheless, the exact mechanism of MSM on attenuating the markers of oxidative stress is not well established and further exploration is needed.
